# High Prevalence of Sarcopenia in HIV-Infected Individuals

**DOI:** 10.1155/2018/5074923

**Published:** 2018-07-12

**Authors:** Patricia Echeverría, Anna Bonjoch, Jordi Puig, Carla Estany, Arelly Ornelas, Bonaventura Clotet, Eugenia Negredo

**Affiliations:** ^1^Lluita contra la Sida Foundation, Hospital Universitari Germans Trias i Pujol, Badalona, Spain; ^2^Universitat Autònoma de Barcelona, Catalonia 08916, Spain; ^3^Statistics and Operations Research, Technical University of Catalunya, Barcelona 08020, Spain; ^4^AIDS Research Institute-IRSICAIXA, Institut Germans Trias I Pujol (IGTP), Hospital Universitari Germans Trias i Pujol, Badalona 08916, Spain; ^5^Universitat de Vic-Universidad Central de Catalunya UVIC-UCC, Vic 08500, Spain

## Abstract

**Background:**

Sarcopenia is a geriatric syndrome that leads to a loss of functionality and mortality.

**Methods:**

We assessed the prevalence of sarcopenia in HIV-infected patients attended in our HIV Unit who had at least two DXA scans from 2000 to 2016 (1,720 DXA scans from 860 individuals). Sarcopenia was determinate according to appendicular skeletal muscle mass index (ASM) calculated as the ratio between skeletal muscle mass index (SMI) by DXA and height^2^ (kg/m^2^). We stratified patients by gender and age (<40, 41-50, and >50 years) and according to the interval between DXAs (≤3, 3-7, 7-10, >10 years). The statistical analysis was performed using SPSS version 19.

**Results:**

Median (IQR) age was 52 (47; 57) years, and 76% were male. The median (IQR) time with HIV infection was 8 (3; 15) years. The prevalence of sarcopenia was 25.7% (95% CI, 22.8-28.7), more prevalent in those aged >50 years (27.8%). Stratifying by gender, 43% of women aged >50 years presented sarcopenia compared with 8.8% of men. The frequency of sarcopenia increased from 37.6% to 49.4% when interval between DXA was 7-10 years (n=109), significantly higher in women than in men (p=0.016). In addition to the traditional risk factors, time with HIV infection was associated with sarcopenia [RR 1.780 (95% CI, 1.314-2.411), p=0.001].

**Conclusion:**

The prevalence and progression of sarcopenia in HIV-infected patients were high, mainly among women. Further studies are necessary to assess the best approaches to prevent this condition and its consequences.

## 1. Introduction

The enormous advances in antiretroviral treatment in the last two decades have made infection by human immunodeficiency virus (HIV) in developed countries to be considered a chronic disease. However, as a consequence of the improvement in survival, the prevalence of comorbidities is increasing, as shown by data from large observational cohorts of HIV-infected patients [1–3]. In addition, some studies suggest that HIV-related chronic inflammation and long-term exposure to some antiretroviral drugs are responsible for accentuated aging in this population [4]. Consequently, physicians are increasingly faced with previously unrecognized comorbid conditions in HIV-infected patients [1–3, 5].

There is increasing evidence that the higher prevalence of bone fractures in HIV-infected individuals may lead to immobility, dependency, and increased morbidity and mortality [6–10]. Although increased bone mass loss is a crucial factor for bone fractures, the presence of sarcopenia (from the Greek* sarx*, or flesh, and* penia*, loss) could contribute to an increased risk of falls and fractures (directly or increasing the frailty) [11].

Sarcopenia is a syndrome characterized by progressive and generalized loss of skeletal muscle mass and strength that has been associated with a risk of adverse outcomes such as physical disability, poor quality of life, morbidity, and death. It is highly prevalent in older populations [12, 13].

The European Working Group on Sarcopenia in Older People (EWGSOP) developed a practical clinical definition and consensus diagnostic criteria for age-related sarcopenia [14]. Previous cross-sectional studies in Europe and North America have described a sarcopenia index (muscle mass/height^2^) and used a cut-off based on two standard deviations (2SD) below mean of young adults [15, 16]. However, sarcopenia definition is currently a matter of debate and several new indices are being tested [14–19].

The most widely used imaging techniques for estimating muscle mass and lean body mass are computed tomography (CT), magnetic resonance imaging (MRI), and dual-energy X-ray absorptiometry (DXA) [14, 15]. DXA is an attractive alternative method for research and for clinical use in the analysis of body composition, providing assessment and quantification of fat mass, lean mass, and bone mineral content, especially through the assessment of nonbone lean mass parameters, such as appendicular skeletal lean mass (ASM) adjusted for body mass index (BMI) and height (ASM/BMI and ASM/ht^2^). ASM, which is a sum of the muscle mass of the arms and legs, is generally used for the skeletal muscle mass index and distinguishes individuals with physiological loss of muscle mass from those with pathological worsening of sarcopenia [14–16].

The multiple factors that contribute to sarcopenia include the aging process, suboptimal diet and bed rest, sedentary lifestyle, chronic diseases, and certain drug treatments [13, 14].

Considering the high relevance of sarcopenia in elderly people, this study aims to determine the prevalence of sarcopenia (determinate by DXA scan) and its progression in a cohort of HIV-1-infected subjects. The study also assesses the relationship between sarcopenia and other HIV-related characteristics and clinical factors.

## 2. Methods

### 2.1. Study Design and Population

This analysis was based on DXA scans from all HIV-infected individuals attended in the HIV Unit of a tertiary hospital who had at least two DXA scans over the last 17 years (from 2000 to 2016). The DXA scans (LUNAR DPX-L, Madison, WI, USA) were requested as part of the patient's follow-up in clinical practice or in the context of clinical trials, and since 2015, DXA scans were requested according to the current recommendations for HIV-infected persons: before starting antiretroviral treatment, men aged >50 years old, postmenopausal women, persons with a history of fractures, and patients with diseases associated with a decrease in bone mineral density. [20]

Sarcopenia was determinate according to appendicular skeletal muscle mass index (ASM) calculated as the ratio between skeletal muscle mass index (SMI) by DXA and height^2^ (kg/m^2^) [15]. The cut-off point used was two standard deviations (2SD) below the mean SMI of young male and female reference groups. Patients were considered to have sarcopenia when the SMI was 5.5 kg/m^2^ in women and 7.26 kg/m^2^ in men [14, 15].

### 2.2. Study Endpoint

The prevalence of sarcopenia was determined in the overall population and stratified by gender and age (<40, 41-50, and >50 years).

Progression of sarcopenia was defined as the difference between the baseline prevalence registered in the first DXA scan and the prevalence calculated in the last DXA scan available for each patient. Patients were stratified according to gender and age, taking into account the interval between the first and the last DXA scan (<3 years, 3-7 years, 7-10 years, and >10 years).

The factors assessed to determine the predisposing risk factors for sarcopenia were gender, age, BMD, body mass index (BMI), specific HIV-related characteristics (time since diagnosis of HIV, cumulative exposure to antiretroviral treatments, CD4 cell count, and viral load) and other clinical parameters (cholesterol levels, osteopenia/osteoporosis), adjusted for gender and age.

Sociodemographic data, HIV-related data, and anthropometric and clinical data were obtained from the medical records.

### 2.3. Statistical Analysis

Demographic and clinical parameters were expressed as the mean and standard deviation (SD) or as the median and interquartile range (IQR); qualitative variables were expressed as frequencies and percentages. Normally distributed continuous variables were compared using the* t*-test; nonnormally distributed variables were compared using the Mann–Whitney test. The dependent* t*-test for paired samples or the Wilcoxon signed rank test was performed to assess the significance of changes observed over time; proportions were compared using the McNemar test.

Univariate* P* values <0.05 were considered significant.

The statistical analysis was performed using IBM SPSS Statistics for Windows, Version 19.0 (IBM Corp, Armonk, New York, USA).

## 3. Results

A total of 1,720 DXA scans from 860 HIV-1-infected individuals were analysed. At time of the first DXA scan, the median (IQR) age of the population was 52 (47; 57) years; men accounted for 76% of the study population. The median (IQR) time since the diagnosis of HIV infection was 8 (3; 15) years, and the cumulative exposure to antiretroviral treatment was 8 (3; 14) years. Almost all patients (94%) had an HIV-1 RNA viral load ≤50 copies/ml and a median (IQR) CD4 T-cell count of 654 (472; 874) cells/mm^3^ (Table 1).

### 3.1. Prevalence of Sarcopenia

The prevalence of sarcopenia overall (according to the parameters evaluated in the DXA scans included in the analysis) was 25.7% (95% CI: 22.8-28.7) (Table 2).

Sarcopenia was more prevalent in patients aged 41-50 years (40%) and >50 years (59%) (Table 2).

When sarcopenia was assessed according to age and gender, 43% of women older than 50 years and 8.8% of men had sarcopenia (p=0.001) (Table 2).

The percentage of sarcopenia in patients with osteopenia and osteoporosis was 34% and 10.5%, respectively. Sarcopenia was not observed in patients with a normal BMD (55%) (p=0.001).

### 3.2. Progression of Sarcopenia and Risk Factors

The frequency of sarcopenia increased from 37.6% to 49.4% (p=0.001) in patients with an interval of 7-10 years between DXA scans (n=109 patients) and from 22% to 25.4% (p=0.046) in those with an interval of more than 10 years between DXA scans (n=209).

When the progression of sarcopenia was determined taking into account gender, the overall prevalence increased from 56% to 66% in women (p=0.016) and from 26.7% to 29.7% in men (p=0.057), with significant differences between the genders (p=0.001).

When age was taken into account, the percentage of sarcopenia in individuals older than 50 years increased from 43% to 52% in women (p=0.001) and from 8.8% to 9% in men (p=0.422). The difference between the genders was significant (p=0.001).

The univariate analysis showed that sarcopenia was more prevalent in women than in men [RR 4.502 (95% CI: 3.227-6.281), p=0.001], in patients with osteopenia [RR 0.415 (95% CI: 0.303-0.568), p<0.001], and in patients with osteoporosis [RR 0.237 (95% CI: 0.151-0.374), p<0.001].

Multiple regression analysis adjusted for age and gender revealed associations between sarcopenia and other clinical and epidemiological variables included in the analysis (Table 3), such as time with HIV infection [RR 1.780 (95% CI: 1.314-2.411), p=0.001]. However, no associations were observed between HIV-RNA viral load >50 copies/mL and sarcopenia [R 0.805 (95% CI: 0.610-1,062), p=0.125], neither between CD4 T-cell counts and sarcopenia [R 0.816 (95% CI: 0.568-1,172), p=0.271].

## 4. Discussion

In our study, sarcopenia affected one-fifth of the population and significantly increased over time, being more prevalent in women older than 50 years. Longer duration of HIV infection was associated with a greater risk of sarcopenia.

Based on DXA scans, we found a high prevalence of sarcopenia in HIV-infected individuals (25%). This finding is similar to rates reported in longitudinal and other cross-sectional studies in HIV-infected persons after adjusting for age, gender, and BMI [11, 21].

Ferreira Da Silva* et al.* [11] evaluated the presence of presarcopenia and sarcopenia in HIV-infected individuals and compared a group of virologically suppressed patients receiving regular antiretroviral therapy and elderly non-HIV-infected controls. Consistent with our results, the authors showed a strong positive association between presarcopenia and sarcopenia in persons with HIV infection, even after taking into account the higher mean age of controls (59 versus 70 years) and adjusting for age and BMI.

Similarly, Wasserman* et al.* [21] reported a prevalence of low muscle mass similar to ours (between 18.8% and 21.9%, depending on the definition used) in midlife and older HIV-infected individuals, particularly men, despite CD4 cell reconstitution and viral suppression. However, in our analysis, when we considered only males, we found a lower prevalence of sarcopenia. [11, 21] Yarasheski* et al.* [22] found similar data in a longitudinal study that also included a non-HIV-infected control group.

The small sample size of the previous studies, however, makes it difficult to stratify patients by gender and age. Our large sample, on the other hand, enabled us to assess differences between men and women of different ages and identify a higher prevalence among women, mainly those older than 50 years.

The prevalence of sarcopenia clearly varies according to which diagnostic criteria are applied to different study samples.

As expected, progression of sarcopenia was more pronounced in patients with an interval of ≥7 years between DXAs. Women aged ≥50 years presented more severe sarcopenia. Although data on progression are comparable to findings for other HIV-infected cohorts [11, 21–23], no previous studies have taken gender into account.

Our results confirm that the traditional risk factors for sarcopenia in HIV-infected population are the same as in the general population. Older age, female gender, and low BMI and low cholesterol were associated with low muscle mass [22]. In addition to the traditional risk factors, however, other HIV-related factors could play an important role in the development of sarcopenia in this population. As our study shows, longer time with HIV infection predisposed to muscle mass loss, thus potentially explaining the higher prevalence than in noninfected controls, as confirmed in the previously mentioned studies (20% in HIV-infected persons versus 11%-28% in non-HIV-infected persons). In contrast, CD4 cell count and viral suppression were not associated factors. The fact that most patients had an undetectable viral load prevented us from assessing the role of viral replication on muscle mass loss.

The retrospective design of our study was not possible to determine the impact of some risk factors (as smoking, alcohol use, physical activity, or antiretroviral treatment) on the development of sarcopenia, due to the lack of necessary information on the medical report. Prospective studies are necessary (which included a non-HIV-infected control group) to compare the prevalence of sarcopenia between HIV-infected subjects and non-HIV population.

Although we were able to draw robust conclusions especially in postmenopausal women owing mainly to the huge number of DXA scans (1,720 scans from 860 HIV-infected individuals), the wide age range (with 507 patients aged ≥50 years), and the high rate of women included (a quarter of the population).

Therefore, given the prevalence and progression of sarcopenia over time and sarcopenia-associated consequences such as fragility, disability, and mortality [11, 21–23], physicians should consider this syndrome in vulnerable persons, mainly postmenopausal women, to prevent progression and its clinical consequences.

In summary, this exploratory analysis shows a high prevalence of sarcopenia among HIV-infected persons, particularly women, and a positive association between sarcopenia and time with HIV infection. Future research should determine the impact of sarcopenia on morbidity, physical function, and quality of life in HIV-infected individuals and develop approaches to prevent the condition.

## Figures and Tables

**Table 1:** Clinical and epidemiological characteristics of the study population at time of the first DXA scan.

	**N=860**

Age, years (median [IQR])	52 (47; 57)

Age ≥50 years (%)	59

Gender (male) (%)	76

Time since diagnosis of HIV infection, years (median [IQR])	8 (3; 15)

Cumulative exposure to antiretrovirals, years (median [IQR])	8 (3; 14)

CD4 cell count/*μ*L (median [IQR]) (baseline)	552 (377; 728)

HIV-RNA ≤50 copies/mL (%)	94

Osteopenia/osteoporosis (%)	51 / 18

Body mass index (median [IQR])	23 (21; 25)

Total cholesterol (mmol/ml) (median [IQR])	4.7 (4; 5.7)

DXA (dual-energy X-ray absorptiometry).

**Table 2:** Prevalence of sarcopenia in HIV-infected persons.

**Prevalence of sarcopenia according to gender**						

	**First DXA **			**Last DXA **		

	**Female ** ^**∗**^ ** ** (n=210)	**Male ** ^**∗∗**^ (n=650)	**P value**	**Female ** ^**∗**^ ** ** (n=210)	**Male ** ^**∗∗**^ ** ** (n=650)	**P value**

No sarcopenia (%)	35	70	<0.001	43	73	<0.001

Sarcopenia (%)	65	30	<0.001	57	27	<0.001

**Prevalence of sarcopenia according to age**						

≤40 years (%)		1				

41-50 years (%)		40				

>50 years (%)		59				

**Prevalence of sarcopenia according to gender and age ≥50 years**						

**N=507**	**Female ≥50 years ** n=129			**Male >50 years ** n=378		

Sarcopenia n (%)	55 (43)			33 (8.8)		

^**∗**^ P value in women between first DXA and last DXA (p=0.001);^**∗∗**^ P value in men between first DXA and last DXA (p=0.057).

**Table 3:** Multivariate adjusted logistic regression analysis.

	**Adjusted** ^**a**^	

	**RR (95% CI)**	**P value**

Age (30-50 years)	3.26 (0.928-11.505)	<0.001

Gender (female vs male)	4.502 (3.227-6.281)	<0.001

Time since diagnosis of HIV infection >5 years	1.780 (1.314-2.411)	< 0.001

Body mass index >20	0.194 (0.076-0.497)	<0.001

Total cholesterol <140 mg/dL	1.468 (0.949-2.271)	<0.001

^a^The final model of multivariate analyses was adjusted for the following predictors: age, gender, time of diagnosis of HIV-infection >5 years, BMI >20, viral load ≥50 copies/mL,and total cholesterol <140 mg/dL.

## Data Availability

This data is available in the following link: http://www.natap.org/2017/AdverseReactComor/AdverseReactComor_15.htm. The data used to support the findings of this study are available from the corresponding author upon request.
